# State Medical Societies

**Published:** 1879-04-20

**Authors:** 


### *STATE MEDICAL SOCIETIES.*

Georgia Medical Association, at Rome Ga., April 16th, meets about the time we go to press, and we cannot therefore, note its proceedings in the present issue.

The Kentucky State Medicai Society meets at Danville, May 13th, 1879.

Colorado Medical Society, at Colorado Springs, Tuesday, May 13, 1879. Dr. A Stedman, Denver, President; Dr. C. C. Lathrop, Denver, Permanent Secretary.

State Medical Society of Arkansas, in Little Rock, Wednesday, May 7, 1879. Dr. A. A. Horner, Helena, President; Dr. R. G. Jennings, Little Rock, Secretary.

Ohio State Medical Society, in Dayton, June 3, 1879. Dr. B. B. Leonard, West Liberty, President; Dr. J. F. Baldwin, Columbus, Secretary.

Medical Society of the State of North Carolina, in Greensborough, Tuesday, May 20, 1879. Dr. Charles Duffy, Jr., Newbern, President; Dr. L. J Picot, Littlejohn, Secretary.

Medical Society of the State of West Virginia, in Martinsburg, June 4, 1879. Dr. Wesley H. Sharp, Volcano, President; Dr. M. F. Hullihen, Wheeling, Secretary.

